# The Prevalence of Endoparasites in Stray Cats in Western Iran

**Published:** 2019-04

**Authors:** Behrouz EZATPOUR, Seyed Mahmoud Reza TAHERIAN, Saeid BAJALAN, Mohammad Hassan KAYEDI, Hossein MAHMOUDVAND, Khatereh ANBARI

**Affiliations:** 1. Razi Herbal Medicines Research Center, Lorestan University of Medical Sciences, Khorramabad, Iran; 2. Department of Public Health, School of Health, Lorestan University of Medical Sciences, Khorramabad, Iran; 3. Department of Medical Parasitology and Mycology, School of Medicine, Hamadan University of Medical Sciences, Hamadan, Iran; 4. Department of Social Medicine, Medical School, Lorestan University of Medical Sciences, Khorramabad, Iran

## Dear Editor-in-Chief

Domestic and wild animals particularly rodents, stray dogs and stray cats play a key role in preserve zoonotic parasitic infections under natural conditions, and they act as reservoir hosts of human infections. Cats can be act as definitive hosts for many parasite infections, such as toxoplasmosis, opisthorchiidosis, toxocarosis and others. Cats by hunting and eating of rodents an important source for cat helminthes infection- are considered to be either intermediate or paratenic hosts in their life cycles of some gastrointestinal parasites. The defecating habit (normally hide under ground) of cats facilitates dissemination of parasites to humans, particularly in public places such as parks.

There are reports on the prevalence, species composition and burden of parasites in cats from different parts of Iran, and revealed overall prevalence of 88.5 % to 97.3% of parasites (1–4). However, there is shortage of such information in some areas of the country including western Iran.

Therefore, the present study was designed to identify the species and degree of infection of intestinal parasites affecting cats in Khorramabad, in west of Iran. Its attitude from free seas is 1125 m and surrounded by mountains. The temperature mean value of this city is 17.60 C, however, during the cooler periods (November and March) the average temperature drops to about 5 C, relative humidity mean value is 46.08% and precipitation mean monthly value is 42.74 mm (5).

This cross-sectional study was performed between Apr to Jun 2014. A total of 124 stray cats (70 males, 54 females) were captured in five geographic districts (East, West, North, South and Central) of city. The stray cats were captured with baited cage traps. Calculation of age was performed based on body size, dental development and maturation of genital structure. Fecal samples were collected in labeled plastic bags and transported to parasitological laboratory, for processing within 12 h, using the direct smear, floatation and formol - ether techniques.

**Fig. 1:**
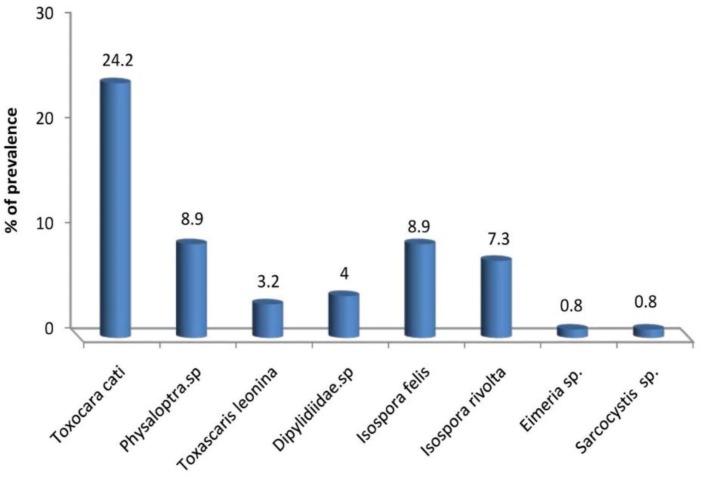
The prevalence of parasites in cats of Khorramabad, Iran

**Fig. 2:**
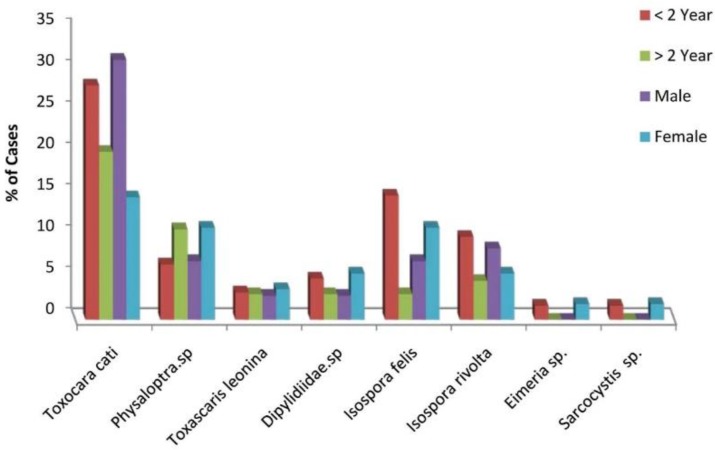
The prevalence of parasites in cats (n=124) of different age groups and sex in Khorramabad, Iran

Helminthes eggs and oocysts of at least one of the eight parasites were reported from 50.8% of the feline fecal samples examined. Results are shown in Fig. 1 and 2. Helminthes eggs were more frequently found in fecal samples than protozoa. The established parasite fauna consisted of three nematodes (*Toxocara cati, Physaloptra* spp. and *Toxascaris leonine*), four coccidian (*Isospora felis, I. rivolta, Eimeria* spp*. and Sarcocystis* spp.) and one cestodes (Dipylidiidae spp.). *T. cati* was the most frequently recorded parasite (24.2%). *Physaloptra* spp. and *T. leonina* were two further nematodes detected in a prevalence of 11% and 4%, respectively. Parallel infection with two species was observed in 18 (14.5%) of the stray cats. The prevalence of *Sarcocystis* spp. infection in stray cats was 1%. General endoparasite infection was detected in 50.8 % of cats. These results correspond with the prevalence of 55.8 % from Greece (6), and higher than a study in Mexico (45%) (7). These differences may be due to environmental, regional or climatic variations. The present investigation found *T. cati* as the most prevalent species (24.2%) (Fig. 1), correlates with a study in Mashhad (28.8%) (2).

Researchers explained the impact of drying up and sunlight decrease on the ability of infection of oocyst and eggs larvae (8). Environmental and climatic factors have significant importance in prevalence of helminthic parasites, since eggs are poorly resistant to high temperatures and low air relative humidity. Although, nowadays-close relationship between cat and human in the world, but regarding to the Iranian cultural and religious believes, having pet is less common. On the other hand, people with occupations requiring contact with soil in environments frequented by cats are considerably more probably to contract with parasites mentioned above.
